# Providing Experience for Undergraduate Nursing Students to Care for Older Adults: A Qualitative Study

**DOI:** 10.1093/geroni/igaa057.1782

**Published:** 2020-12-16

**Authors:** Christine Pariseault, Christina Whitehouse, Melissa O’Connor

**Affiliations:** Villanova University, Villanova, Pennsylvania, United States

## Abstract

Care of the older adult can be complex and frequently influenced by ageism. Nursing students do not have the frequent opportunity to provide care for older adults. The purpose of this pilot study was to expose sophomore nursing students to older adults earlier and more often in the undergraduate curriculum by providing a unique clinical experience at St. Thomas of Villanova Monastery, a residential facility for retired Augustinian priests. This study examines the experience of students’ participation in this clinical experience. Qualitative content analysis of 12 student logs was conducted. Themes that emerged included: age-related changes, environmental considerations, psychosocial needs and changes, and consideration of gerontology as a career choice and existing bias. Students gained a valuable understanding of the unique age-related changes that older adults are experiencing. Early experiences are vital in the curriculum and provide enhanced engagement in gerontology.

